# Evolution of Senescence by Damage Accumulation That Accelerates With Age Throughout an Organism's Lifespan

**DOI:** 10.1002/ece3.72988

**Published:** 2026-02-25

**Authors:** Darar Bega, Lilach Hadany

**Affiliations:** ^1^ School of Plant Sciences and Food Security Tel Aviv University Tel Aviv Israel

**Keywords:** aging, Gompertz—Makeham mortality, negligible senescence, Peto's paradox, Strehler—Mildvan correlation

## Abstract

In recent years, senescence is increasingly understood as a process of damage accumulation that accelerates with age throughout an organism's lifespan. That understanding has rarely been introduced to senescence evolution theory. In classic models, including Mutation accumulation and Antagonistic pleiotropy, the intensity of selection over genes is determined by the timing of their effect on mortality. They conclude senescence evolution occurs because of weak selection on late‐acting genes. Here we explore, consistent with recent evidence, an alternative model: where genes affect mortality throughout an organism's lifespan, and the shape of this effect determines selection. We expanded Hamilton's classic model of senescence evolution using these notions. Our model takes into account evolutionary dynamics between external mortality risk, potential mortality risk from internal damage, reproduction start age, and reproduction rate. The analysis of the model suggests biological limitations on reducing the potential mortality risk from internal damage can lead to a positive feedback loop in senescence evolution where genes that slow senescence can increase selection for further senescence retardation. Our model sheds light on several phenomena, not fully explained by classic theory, including Peto's paradox, Strehler—Mildvan correlation, and negligible senescence.

## Graphical abstract

Senescence evolution is usually explained by reduced selection on late acting genes. Here we develop a new model for senescence evolution that complements the classic theory by focusing on genes that affect mortality throughout lifespan. Our work sheds light on the dynamics of senescence evolution and helps to reconcile phenomena that presented challenges to the classical theory: negligible senescence, Peto's paradox, and Strehler—Mildvan correlation.

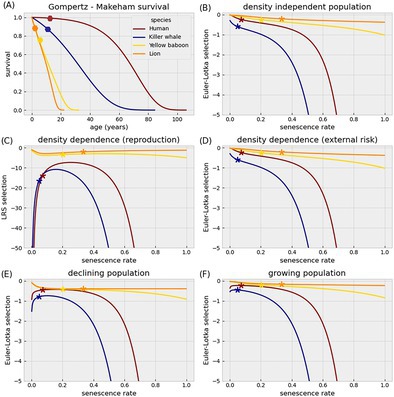

## 1 Introduction

At first glance, senescence evolution appears paradoxical. It is hard to imagine how physical deterioration could be adaptive, and if it is not, why was it not selected against? The main explanation suggested in the literature is that senescence is not adaptive, but rather evolves because the reproductive value deteriorates with age. The reproductive value estimates the expected genetic contribution of an individual to future population (Fisher 1999). Genes that reduce mortality at advanced ages will carry a smaller advantage than genes reducing mortality at younger ages, because a portion of the reproduction was already achieved. This decrease in the strength of selection with age can lead to the removal of senescence delaying genes from the population, due to either drift or detrimental pleiotropic effects (Medawar 1952; Williams 1957). Hamilton (1966) was the first to formulate expressions for the strength of selection on senescence manipulating genes. He considered genes that change mortality by a constant, either instantaneously or permanently, starting at a time interval after maturity.

Despite the success of these classic explanations, several phenomena have not been fully addressed. One is the existence of species exhibiting negligible senescence—mortality rate that remains constant with age (Jones and Vaupel 2017; Flatt and Partridge 2018). Moreover, these species often exhibit high potential mortality risk from internal damage accumulation (e.g., body size in whales, oxidative pressure in naked mole‐rats, see discussion), which has been predicted to reduce the reproductive value for advanced ages (Williams 1957). In fact, the opposite correlation—negative association between senescence rate and potential internal mortality risk—was observed between populations of multiple species (Strehler and Mildvan 1960; Golubev 2019; Shen et al. 2017). Also not aligned with expectations from these classic explanations, recent empirical evidence suggests that many senescence manipulating genes do so from birth, throughout an organism's lifespan (Maklakov et al. 2015; Kinzina et al. 2019; Kerepesi et al. 2021). For example, by‐products of breathing are free radicals. These are highly active reductive agents that react with DNA, proteins, and lipids to cause metabolic mistakes (Sitte and von Zglinicki 2003). The genes that repair these mistakes are active from conception. Comparative evolution studies show that regulation of oxidative damage repair is associated with longevity in different organisms, for example, in rodents (Csiszar et al. 2007; Pérez et al. 2009; Gorbunova et al. 2014). The same is true for accumulation of somatic mutations, and other types of damage (Cagan et al. 2022; López‐Otín et al. 2023).

If not repaired or replaced, many types of damage can accumulate (López‐Otín et al. 2023). With time, mechanisms of damage repair, protection, and replacement themselves can be damaged and lose efficiency (Hernando et al. 2021). Thus, a pattern of exponential increase in many types of damage, and in biological systems vulnerability to failure, is expected (Ledberg 2020). Indeed, when plotting mortality with age on a logarithmic scale a straight line is produced at advanced age (Finch 1994; Kirkwood 2015). The slope of that line is referred to as the rate of senescence (Finch 1994; Rozing and Westendorp 2008). Early‐life mortality is dominated by pre‐development external risks and parental deleterious alleles, masking the relatively modest contribution of senescence (Kinzina et al. 2019; Siler 1979). The Gompertz—Makeham function (Gompertz 1997; Makeham 1860) describes mortality μ as a function of age x. It has an age dependent part and an age independent part: $\mu_{x}=Ge^{\mathrm{Rx}}+M$ where G and M are constants and R is the rate of senescence. Several models showed that damage accumulation gives rise to Gompertz—Makeham like mortality functions under certain conditions (Box 1). These mechanistic models explain mortality patterns very well, but the evolutionary forces that might arise from genes that manipulate their parameters have hardly been explored (but see suggested alternative effects to Hamilton's senescence manipulating genes (Maklakov et al. 2015; Charlesworth 2001)).

BOX 1 **How Damage Accumulation Can Lead to Gompertz – Makeham Mortality.**

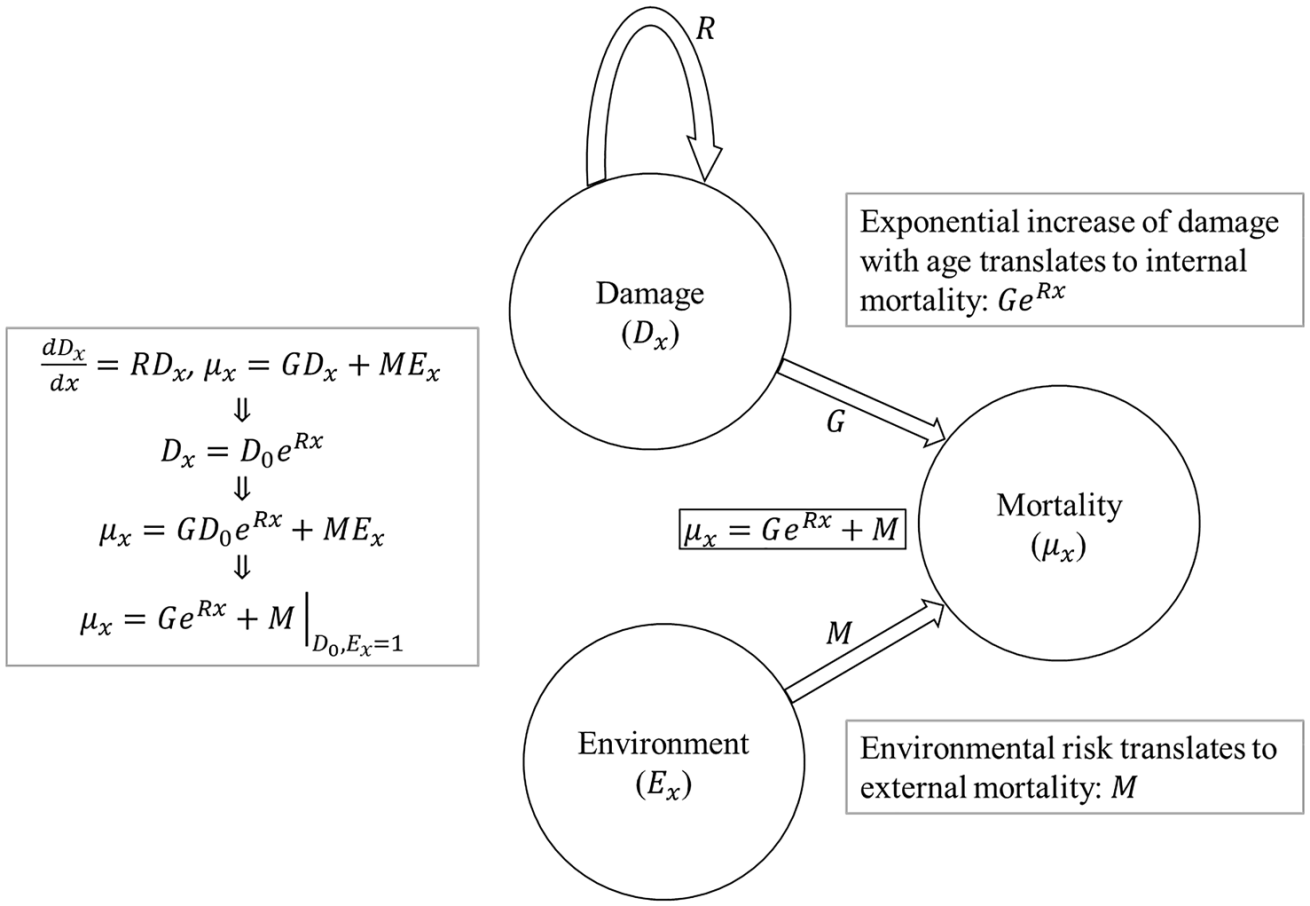

An illustration demonstrating the relationship between damage accumulation and the Gompertz – Makeham mortality model, $\mu_{x}=Ge^{\mathrm{Rx}}+M$. where, G, R, and M are the potential mortality risk from internal damage, senescence rate, and external mortality risk, respectively. Damage induces faster damage accumulation by harming damage repair mechanisms, leading to exponential increase of internal mortality risk with age. Age‐independent mortality risk (external risk, e.g., weather events) adds the Makeham term to the mortality risk function. Notice that G, R, and M can be independent under this simple model assumptions. Charlesworth (2001) showed how a Gompertz function can evolve, given Hamilton's model, from a mutation selection balance. However, Wachter et al. (2013) showed that this result is sensitive to the model's linear approximation of feedback between mutation and selection. A mechanistic explanation was suggested by Gavrilov and Gavrilova (1991) that used reliability theory (Rausand and Hoyland 2003) (developed to predict machines failure rate). When plenty of redundancy exists in a system, where one component failure can cause the failure of the system, the failure hazard rate changes with the age of the system and will asymptotically follow a Gompertz – Makeham function. In addition, general models of damage accumulation often give rise to mortality functions that after reparameterization becomes Gompertz – Makeham like (Ledberg 2020).

To further our understanding of senescence evolution considering these ideas, we expand Hamilton's model to include mutations that affect senescence as a process that starts at birth and increases mortality according to a Gompertz—Makeham function. We first examine selection on senescence rate throughout lifespan as a function of external mortality risk, potential internal mortality risk, reproduction start‐age, and reproduction rate. We then test the possibility of negligible senescence evolving from senescing organisms. Our model includes ecological context: population growth/decline, density dependence, and life‐history trade‐offs. This allows us to predict evolutionary feedback on senescence rate and find an evolutionary stable strategy (ESS).

## 2 Model

First, we define mortality at age x using Gompertz—Makeham (Strehler and Mildvan 1960; Golubev 2004, 2009; Gavrilov and Gavrilova 2005) as the instantaneous rate, $\mu_{x}$, at which death occurs in a very large population: $\mu_{x}=Ge^{\mathrm{Rx}}+M$, where M is the external mortality, not dependent on age, G is a coefficient representing potential mortality risk from internal damage accumulation, and R is senescence rate, which is the rate of an exponential process of damage accumulation, see (de Souza 2022) for notation alternatives. We assume the function parameters represent biological mechanisms that can evolve independently. Notice that when R=0 the organism does not senesce and mortality remains constant with age.

The survival function $l_{x}$ describes the probability of surviving from birth to age x (Stearns 1998): $l_{x}=e^{-\int_{t=0}^{x}\mu_{t}\mathrm{dt}}=e^{-\frac{G}{R}\left(e^{\mathrm{Rx}}-1\right)-\mathrm{Mx}}$. To connect survival to fitness we define a reproduction rate function of age, $m_{x}$, that starts at the age of sexual maturity, such that $x_{s}$ is defined as the reproduction start‐age. We make the simplifying assumption that infant mortality is independent of senescence and ends before reproduction starts. Doing so, we can exclude from the reproduction rate function the proportion of offspring that will die before reproducing from causes that do not relate to senescence (e.g., infant disease), avoiding additional model parameters. We explore two demographic scenarios: when assuming a constantly growing/declining population, and when the population size is rather stable. In the first case, fitness of a growing/declining population can be estimated by the instantaneous growth rate r, which is calculated by solving the Euler—Lotka Equation (1): $1=\int_{x_{s}}^{\infty }e^{-\mathrm{rx}}l_{x}m_{x}\mathrm{dx}$.

Hamilton calculated the change in fitness resulting from a constant increase in $\mu_{x}$ from age a assuming independence from reproduction rate (Hamilton 1966): $\frac{\mathrm{dr}}{{\mathrm{d\mu}}_{\left(a,..,\infty \right)}}=\frac{-\int_{a}^{\infty }\left(x-a\right)e^{-\mathrm{rx}}l_{x}m_{x}\mathrm{dx}}{\int_{0}^{\infty }xe^{-\mathrm{rx}}l_{x}m_{x}\mathrm{dx}}$. Note that for such genes, selection decreases only when a is larger than reproduction start‐age. Thus, senescence is expected to start only after reproductive maturity if genes are selected based on the age of onset of their effect. Several models got pre‐reproduction mortality to affect selection by assuming pleiotropic effects, while keeping the assumptions on the way genes affect senescence (Charlesworth 2001; Kirkwood and Rose 1991). We used a different approach, consistent with recent evidence (Maklakov et al. 2015; Kinzina et al. 2019; Kerepesi et al. 2021): we calculated the change in fitness resulting from a gene that causes an exponential increase in internal mortality from birth (or rather conception). That is, we set a to be 0 and differentiated r with respect to a change in senescence rate, R, instead of a constant increase in $\mu_{x}$ from age *a* (see Appendix S1):

(1) $$\frac{\mathrm{dr}}{\mathrm{dR}}=\frac{\frac{G}{R^{2}}\int_{x_{s}}^{\infty }\left(e^{\mathrm{Rx}}-\mathrm{Rx}e^{\mathrm{Rx}}-1\right)e^{-\mathrm{rx}}l_{x}m_{x}\mathrm{dx}}{\int_{x_{s}}^{\infty }xe^{-\mathrm{rx}}l_{x}m_{x}\mathrm{dx}}$$

When considering a population that is not exponentially growing, but rather fluctuates around a stable size, we estimated fitness using the lifetime reproductive success, *LRS* (Clutton‐Brock 1988): $\mathrm{LRS}=\int_{x_{s}}^{\infty }l_{x}m_{x}\mathrm{dx}\sim 1$. Again, we differentiated both sides of the equation with respect to R. The selection gradient in that case is (see Appendix S1):

(2) $$\frac{\text{dLRS}}{\mathrm{dR}}=\frac{G}{R^{2}}\int_{x_{s}}^{\infty }\left(e^{\mathrm{Rx}}-\mathrm{Rx}e^{\mathrm{Rx}}-1\right)l_{x}m_{x}\mathrm{dx}$$

The differences between Equations (1) and (2) reflect the scenarios they can be used to estimate selection (fitness based on genetic contribution rate versus total). One difference is the denominator in Equation (1), representing the generation time (Charlesworth 1994), where the division by generation time favors shorter lifespans. The other difference is the exponent in Equation (1), $e^{-\mathrm{rx}}$, representing the changing value of reproduction with age, favoring early reproduction in growing population, and late reproduction in declining population.

Density dependence of population growth also influences the suitable fitness estimator (Abrams 1993). When density dependence limits population growth through reproduction control, LRS is the suitable fitness estimator, and when external risk limits population growth Euler—Lotka should be estimated (Metz 1992; Brommer 2000; Roff 2008). We considered four simple cases of density dependence. (a) No density dependence. Populations grow/decline exponentially and selection is estimated by Equation (1). (b) Density dependence through reproduction, assumed to impact all ages equally. For a stable population we get: $m_{x}^{\mathrm{dd}}=\frac{m_{x}}{\int_{x_{s}}^{\infty }l_{x}m_{x}\mathrm{dx}}$, where $m_{x}^{\mathrm{dd}}$ is the reproduction function under density dependence. Such dynamics can be seen in species where competition limits the number of offspring reaching adulthood. Examples include competition for food, mate, and territory. Selection is then estimated by Equation (2). (c) Density dependence through external risk, also assumed to impact all ages equally. Here we get: $M^{\mathrm{dd}}=r+M$, for 0<r, where r is the solution of: $1=\int_{x_{s}}^{\infty }e^{-\mathrm{rx}}l_{x}m_{x}\mathrm{dx}$, and $M^{\mathrm{dd}}$ is external mortality risk under density dependence. Such dynamics can occur when population size increase external mortality risks, as in cases of predation, disease, and self‐produced contaminants. Selection is then estimated by Equation (1). (d) Declining and growing populations were assumed to be limited by reproduction equally for all ages, so $m_{x}^{\text{grow}}=\theta m_{x}^{\mathrm{dd}}$ for 1<θ, and $m_{x}^{\text{decline}}=\theta m_{x}^{\mathrm{dd}}$ for 0<θ<1. For such exponentially growing and declining populations Equation (1) is used for selection estimation.

The reproduction rate function is expected to vary significantly between species. Our model is general and can fit any integrable mortality function, but here we demonstrated, for simplicity, the results in case that reproduction rate is constant from reproduction start‐age, $m_{x}=\zeta$. In this case, fitness optima under trade‐offs do not depend on reproduction rate. An example of model extension to include trade‐offs between life‐history parameters is given by the trade‐off equation: $\left\{\begin{array}{c} R=\left(R_{\max}-R_{\min}\right)z^{\alpha }+R_{\min}\\ \zeta =\left(\zeta_{\max}-\zeta_{\min}\right)\left(1-{\left(1-z\right)}^{\beta }\right)+\zeta_{\min} \end{array}\right.$, where $\left(0\leq z\leq 1\leq \alpha ,\beta \right)$, describing a trade‐off between senescence rate, R, and a reproduction rate, ζ. The parameter z determines both life‐history traits. Adding trade‐offs to changes in life‐history parameters allows the calculation of an evolutionary stable strategy (i.e., ESS). For constant population size under density dependence with respect to reproduction the ESS satisfies $\frac{\text{dLRS}}{\mathrm{dz}}=0$, and $\frac{d^{2}\mathrm{LRS}}{d^{2}z^{2}}<0$ (see Appendix S1). However, this trade‐off is merely an example, and different relationships can exist between each pair of the model parameters ζ, $x_{s}$, G, M, and R. These trade‐offs are more important to the invasion of a new mutation than density induced trade‐off, because a new mutation has minimal effect on the population density.

Here we study the selection forces acting on genes that affect the mortality function including the case of negligible senescence, extend to feedback between selection forces on model parameters, and demonstrate the effect of trade‐offs on senescence rate evolution.

## 3 Results

**FIGURE 1:**
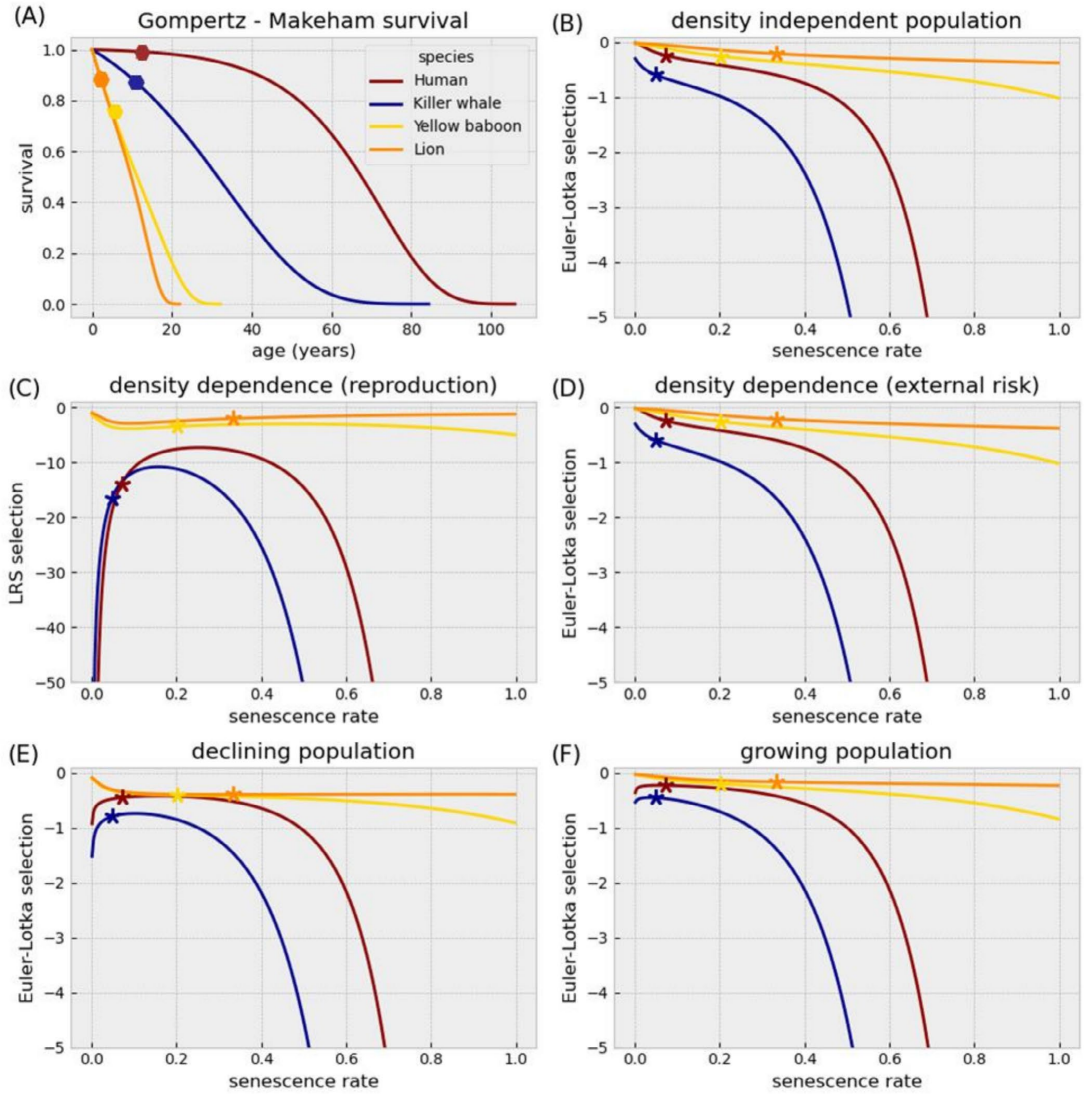
(A) Estimated survival curves (excluding infant mortality) for wild populations of mammals: Red—Human, blue—Killer whale, yellow—Yellow baboon, and orange—Lion. Hexagons represent estimated reproduction start‐age, $x_{s}$, of the mammal species. (B–F) expected theoretical selection on senescence rate changes in wild populations of mammals: Red—human, blue—killer whale, yellow—yellow baboon, and orange—lion. Asterisks represent the observed population senescence rate, R. (B) assuming density independence under Euler—Lotka selection. (C) assuming density dependence through reproduction rate under LRS selection. (D) assuming density dependence through external mortality under Euler – Lotka selection. (E) growing populations under Euler – Lotka selection. (F) declining populations under Euler – Lotka selection. Reproduction rate was altered to cause declining population ($\zeta^{*}=0.1\zeta$), and growing population ($\zeta^{*}=10\zeta$). The Estimated Parameters of species life‐history: Red—human ($G=0.00041,M=0.00001,x_{s}=13,R=0.071$, ζ=0.036), blue—killer whale ($G=0.00094,M=0.00001,x_{s}=11,R=0.048,\zeta =0.051$), yellow—yellow baboon ($G=0.003,M=0.05,x_{s}=5,R=0.208,\zeta =0.182$), and orange—lion ($G=0.0025,M=0.0522,x_{s}=2,R=0.325,\zeta =0.2$).

We explore the differences between our model and classical models in selection for senescence manipulating genes. Most previous analyzes focused on the age of onset of genes effect. We focus instead on genes that affect mortality throughout life by manipulating senescence rate, R. To anchor our exploration in biologically realistic regimes, we used estimated life‐history parameters from data on wild populations of mammals (Jones et al. 2014) (Figure 1A): human ($G=0.00041,M=0.00001,x_{s}=13,R=0.071$, ζ=0.036), killer whale ($G=0.00094,M=0.00001,x_{s}=11,R=0.048,\zeta =0.051$), yellow baboon ($G=0.003,M=0.05,x_{s}=5,R=0.208,\zeta =0.182$), and lion ($G=0.0025,M=0.0522,x_{s}=2,R=0.325,\zeta =0.2$). This variety of life‐histories gives us a realistic parameter range to explore the model dynamics (see Methods for how they were estimated).

### 3.1 Senescence Rate Selection Gradients

We first analyze selection on senescence rate, R, in the simple case without trade‐offs and without co‐evolution between the model parameters. We explore scenarios where fitness is either estimated using population growth rate r (Euler—Lotka) or LRS. We use Euler—Lotka selection for the scenarios of no density dependence, density dependence through external risk and for growing/declining populations. LRS selection is used in the scenario where population size remains constant by density dependence of reproduction rate. In all scenarios, selection on senescence rate grows (becomes more negative) when an already high senescence rate grows such that the age of certain mortality approaches reproduction start‐age (Figure 3B–F). Because lions and yellow baboons have relatively low reproduction start‐age, this pattern is less pronounced for them in the range of senescence rates presented. In a growing population, early reproduction contributes more to fitness than late reproduction, and in declining population the reverse is true. That is why selection on senescence rate is stronger in declining populations (Figure 1E,F). Selection gradients when density dependence works through external mortality is equal to no density dependence because external mortality risk, M, is fully compensated by r in the Euler—Lotka equation. In these scenarios, selection on senescence rate decrease (becomes less negative) with slower senescence rate (Figure 1B,D). However, across demographic and density dependence scenarios selection does not nullify—not even for negligible senescence (Figure 1B–F). This pattern is most pronounced for the killer whale parameterization, where R=0 does not eliminate selection on R (Figure 1B–F). Moreover, in the scenarios that the population grows, declines or under density dependence through reproduction, selection on senescence rate may increase as senescence slows (Figure 1C,E,F). These observations motivate a systematic examination of how selection for negligible senescence varies across the model parameters.

### 3.2 Negligible Senescence Selection

**FIGURE 2:**
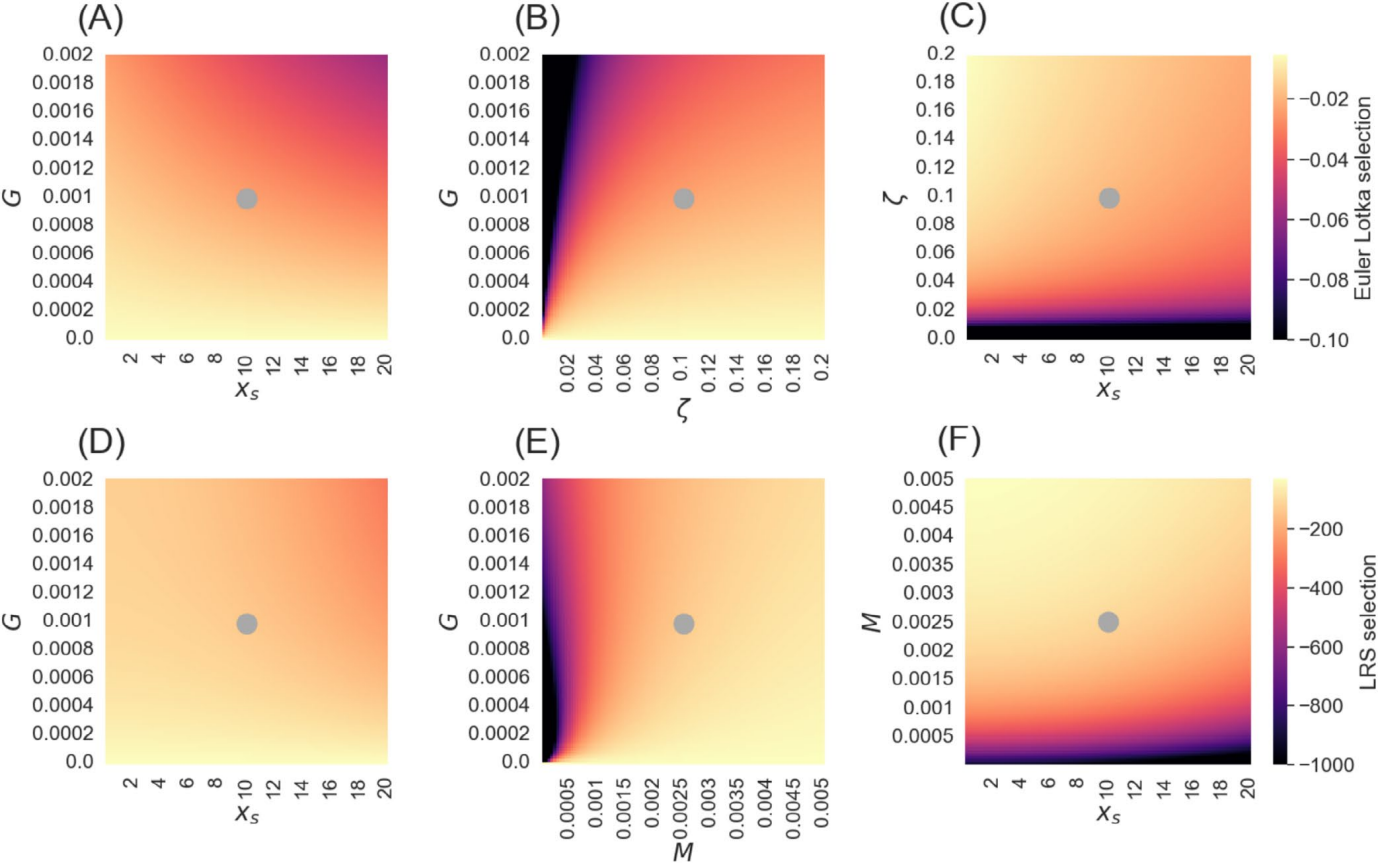
Selection on senescence rate under negligible senescence (see Appendix S1 for derivation of selection gradients under negligible senescence). Color represents selection strength. The results fit an organism with negligible senescence and the parameters: $G=0.001,M=0.0025,x_{s}=10,\zeta =0.1$ marked by a gray dot. (A–C) present Euler—Lotka selection change with the parameters G, $x_{s}$, and ζ. Density dependence through external mortality would not affect these results (see Appendix S1). (D–F) present LRS selection change with the parameters G, $x_{s}$, and M. Density dependence through reproduction rate would not affect these results (see Appendix S1).

We derived equations of selection for negligible senescence under both fitness estimators – r and LRS (see Appendix S1). When fitness is estimated using population growth rate, r, the effect of the parameters are as follows: selection increases with potential mortality risk from internal damage, G, and reproduction start‐age, $x_{s}$, and it decreases with reproduction rate, ζ. It is not influenced however, by external mortality, M (Figure 2A–C). When fitness is estimated using LRS, selection for negligible senescence decreases with external mortality, M, and increases with reproduction start‐age, $x_{s}$ (Figure 2D,F). More complex dynamics exist with risk from internal damage, G. When M is higher than G, selection for negligible senescence can increase with G (if $x_{s}$ is far enough from $x_{d}$), but when M is low compared to G, selection decreases with G (Figure 2E, see Appendix S1 for exact conditions). In other words, the evolution of negligible senescence is more likely when mortality from internal risks is strong compared to external risks, and their total is not too high. Killer whales have exactly these Gompertz—Makeham parameters together with high reproduction start‐age and low reproduction rate, explaining why selection for negligible senescence is relatively strong for their parameterization across all scenarios (Figure 1B–F at R=0). High selection for negligible senescence suggests positive evolutionary feedback on senescence rate.

### 3.3 Positive and Negative Feedback in Senescence Evolution

**FIGURE 3:**
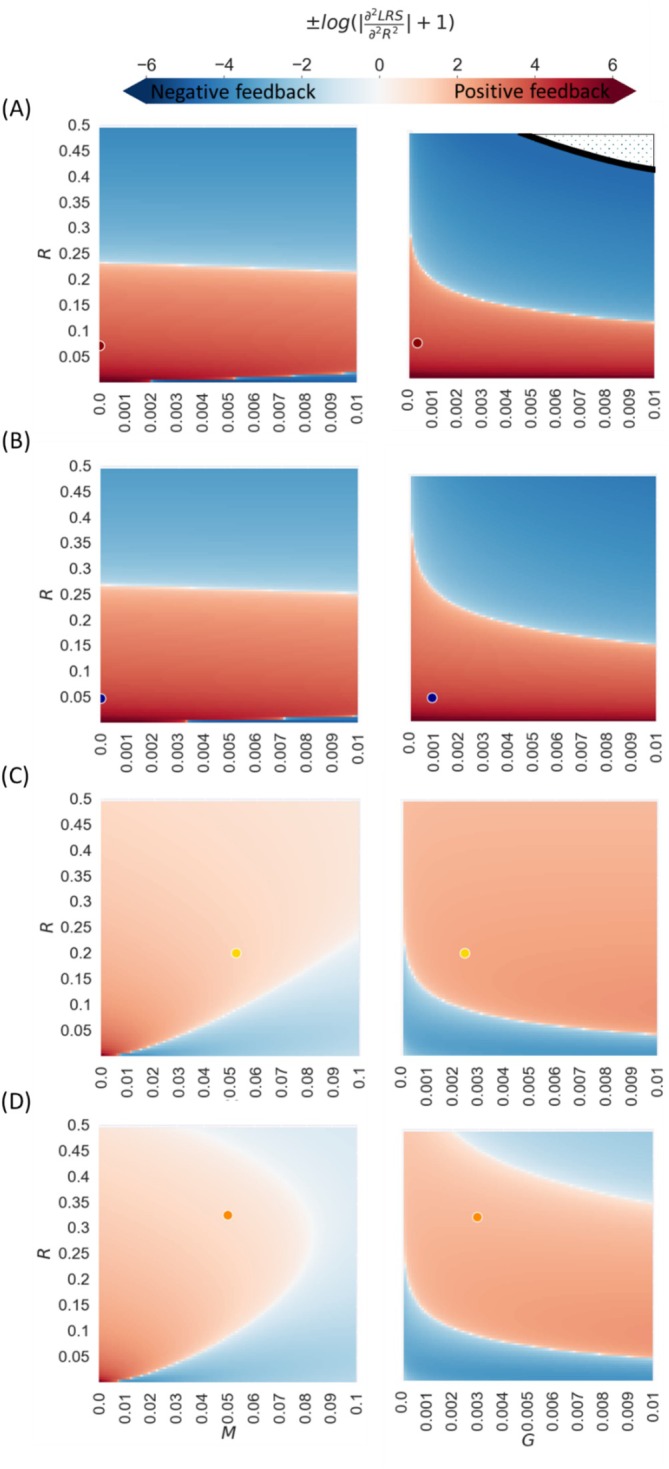
Positive and negative feedback in senescence rate evolution assuming density dependence through reproduction. Color represents the second derivative of the life‐time reproductive success fitness estimator with respect to senescence rate, log‐modulus transformed. Under positive feedback (red) decreasing senescence rate will increase selection for further decrease in senescence rate. Under negative feedback (blue) decreasing senescence rate will decrease selection for further senescence rate decrease. The X‐axes represent potential internal mortality risk from internal damage G (right panels), and external mortality risk M (left panels). Y‐axis represents senescence rate, R. Panels A–D fit different model parameters (G, M, ζ, and $x_{s}$) estimated for wild populations of species (see Results): A—Human, B—Killer whale, C—Yellow baboon, and D—Lion. Dots represents the estimated parameters R and G (right panels)/ *M* (left panels) of the species: Red—Human, blue—Killer whale, yellow—Yellow baboon, and orange—Lion. Dotted white area represents parameter values where survival drops below 0.0001 before reproduction start‐age (fitness is estimated to equal 0).

Population cannot continually grow or decline in evolutionary time scale. Therefore, exploration of evolutionary feedback should include density dependence. From the two fitness estimators LRS and r we hereafter focus on LRS as it covers common density dependence dynamics: through reproduction, offspring recruitment, and juvenile mortality (see Appendix S1 for evolutionary feedback using r, suitable for other scenarios). Feedback on senescence rate evolution is positive if the second derivative of fitness with respect to R is positive ($0<\frac{\partial^{2}\mathrm{LRS}}{\partial^{2}R^{2}}$, $\frac{\partial^{2}r}{\partial^{2}R^{2}}$) and vice versa. In other words, positive feedback occurs when decreasing senescence rate increases selection for further senescence retardation. Applying the equations to the exampled mammal parameter space, we discover complex dynamics between senescence rate, R, potential internal risk, G, and external risk M (Figure 3). Positive feedback when senescence approaches 0 is expected approximately when G is larger than $\frac{M}{2}e^{-0.12x_{s}}$ (e.g., humans and killer whales for low values of R—Figure 3A,B but not in lions and yellow baboons—Figure 3C,D) and positive feedback when R is not close to 0 is expected when $x_{s}$ is far from the age of certain mortality $x_{d}$ (yellow baboons and lions Figure 3C,D). When R is high such that reproduction start‐age is close to the age of certain death, feedback is always negative (when senescence increases, selection for reducing it grows). Evolutionary feedback on senescence rate can also occur through dynamics with other traits.

### 3.4 Co‐Evolution of Senescence With the Other Model Parameters

**FIGURE 4:**
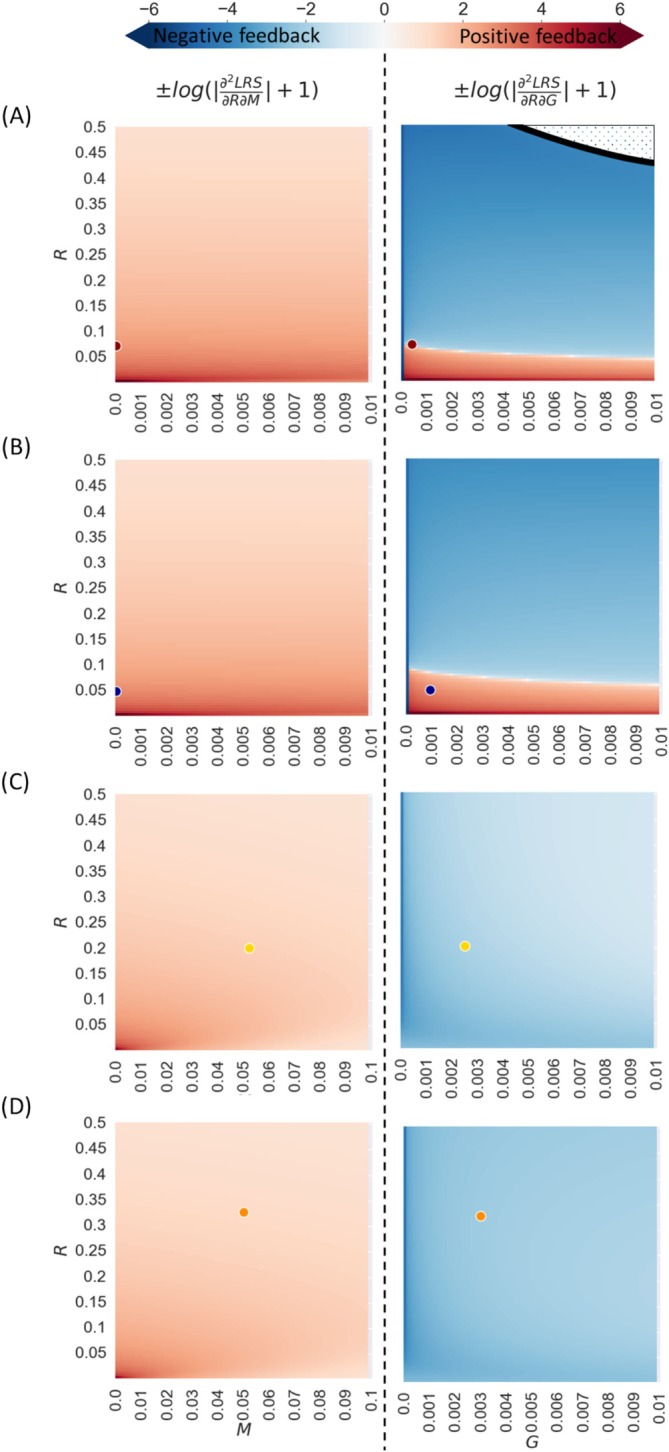
Positive and negative feedback in senescence rate evolution assuming density dependence through reproduction. Color represents the mixed derivative of the life‐time reproductive success fitness estimator with respect to senescence rate and external mortality (left panels)/potential mortality risk from internal damage (right panels), log‐modulus transformed. Under positive feedback (red) decreasing senescence rate will increase selection for further decrease in external mortality (left panels)/potential mortality risk from internal damage (right panels) and vice versa. Under negative feedback (blue) decreasing senescence rate will decrease selection for further external mortality (left panels)/potential mortality risk from internal damage (right panels) decrease. The X‐axes represent potential internal mortality risk from internal damage G (right panels), and external mortality risk M (left panels). Y‐axis represents senescence rate, R. Panels A–D fit different model parameters (G, M, ζ, and $x_{s}$) estimated for wild populations of species (see Results): A—Human, B—Killer whale, C—Yellow baboon, and D—Lion. Dots represents the estimated parameters R and G (right panels)/M (left panels) of the species: Red—Human, blue—Killer whale, yellow—Yellow baboon, and orange—Lion. Dotted white area represents parameter values where survival drops below 0.0001 before reproduction start‐age (fitness is estimated to equal 0).

We now consider genes that influence the other model parameters—external mortality, M, the potential mortality risk from internal damage, G, reproduction start‐age, $x_{s}$, and reproduction rate, $m_{x}$, assuming it is constant ($m_{x}=\zeta$). Unsurprisingly, selection always favors reducing M, G, $x_{s}$, and increasing ζ (see Appendix S1 for their derivation). Selection gradients for all model parameters are continuous, so mixed derivatives are symmetric (e.g., $\frac{\partial^{2}\mathrm{LRS}}{\partial M\partial R}=\frac{\partial^{2}\mathrm{LRS}}{\partial R\partial M}$). The signs of these mixed derivatives identify parameter combinations producing either positive or negative co‐evolutionary feedback between R and the other traits (Figure 4). When G>M and $x_{s}<<x_{d}$, negligible senescence can evolve (e.g., positive feedback for low R in humans and whales Figure 4A,B, but not in lions and baboons Figure 4C,D). However, selection for reducing G remains strong even when senescence rate approaches 0 (see Appendix S1), but not vice versa ($\lim_{G\to 0}\frac{\text{dLRS}}{\mathrm{dR}}=0$). Negligible senescence can thus arise only if a biological limit on lowering G exists. Such limit is expected for most species, since internal damage accumulation can cause mortality in many ways. For example, large body size leads to high potential internal risk of cancer (relatively high G). This might facilitate the evolution of slower damage accumulation during cell division (low R), resulting in slower senescence. This result supports similar explanations to Peto's paradox—the lack of correlation between body size and cancer mortality risk (or why whales don't get cancer?) (Caulin and Maley 2011). When M>G or $x_{s}$ is close to $x_{d}$, negative feedback is expected between G and R. This will result in a Strehler—Mildvan like correlation between species—negative correlation between G and R (Strehler and Mildvan 1960) (the Strehler—Mildvan correlation was observed between different populations within species). Moreover, biological lower bound on G could increase selection for reducing R, reinforcing the same pattern. We summarized the conditions for evolutionary feedback on senescence rate and associated phenomena (Tables 1 and S1 in Appendix S1).

**TABLE 1:** Evolutionary dynamics under LRS fitness estimation.

Parameter evolving	Selection on parameter	Positive evolutionary feedback on R	Approximate simplification of condition	Negative evolutionary feedback on R	Approximate Simplification of condition	Relevance to phenomena
R	$\frac{\text{dLRS}}{\mathrm{dR}}$	$\frac{d^{2}\mathrm{LRS}}{d^{2}R^{2}}>0$	$G>\frac{Me^{-0.12x_{s}}}{2}\;\text{and}\;R\ll 1$ or $x_{s}\ll x_{d}\;\text{and}\;0\ll R$	$\frac{d^{2}\mathrm{LRS}}{d^{2}R^{2}}<0$	$G<\frac{Me^{-0.12x_{s}}}{2}\;\text{and}\;R\ll 1$ or $x_{s}\sim x_{d}$	Senescence evolution, Negligible senescence
G	$\frac{\text{dLRS}}{\mathrm{dG}}$	$\frac{d^{2}\mathrm{LRS}}{\text{dRdG}}>0$	$G>M\;\text{and}\;x_{s}\ll x_{d}$	$\frac{d^{2}\mathrm{LRS}}{\text{dRdG}}<0$	M>G or $x_{s}\sim x_{d}$	Strehler – Mildvan, Peto's paradox
M	$\frac{\text{dLRS}}{\mathrm{dM}}$	Always	—	Never	—	Williams hypothesis
$x_{s}$	$\frac{\text{dLRS}}{dx_{s}}$	Never	—	Always	—	Peto's paradox

### 3.5 Senescence Rate ESS Under Trade‐Off

**FIGURE 5:**
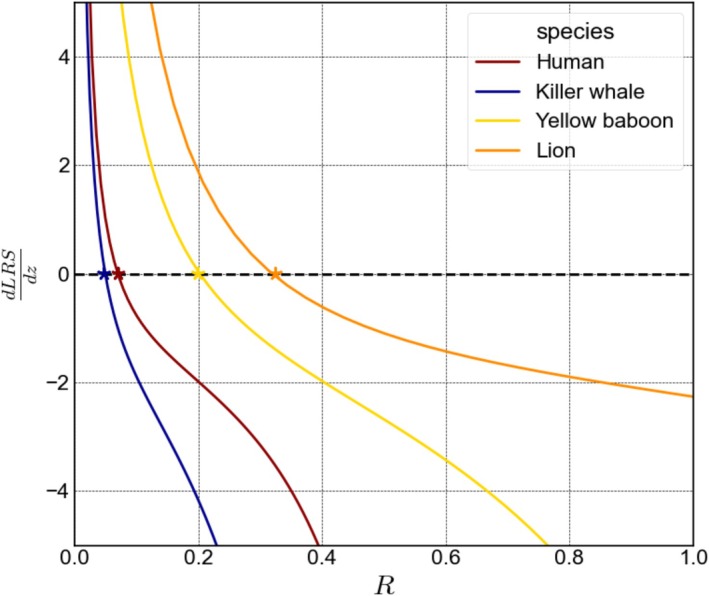
Finding the ESS senescence rate under a trade‐off with reproduction rate. The x‐axis represents senescence rate and the y‐axis represents selection on a gene that changes both senescence and reproduction rates according to their trade‐off. The predicted ESS is obtained where the selection gradient is equal to 0 and the second derivative is negative. Stars represent the estimated senescence rate of the species population. The parameters of the trade‐off are assigned: β=1, $R_{\min}=0.0001$, $\zeta_{\min}=0$. $R_{\max}$ is calculated for each species such that reproduction start‐age must precede death. $\zeta_{\max}$ is calculated for each species given the trade‐off includes the species senescence and reproduction rates. α is brute‐force fitted for every species so the predicted ESS will equal their senescence rate: Human – $\zeta_{\max}=0.36$, α=1.02; whale – $\zeta_{\max}=0.37$, α= 1.25; yellow baboon – $\zeta_{\max}=0.80$, α=1.45; lion – $\zeta_{\max}=1.15$, α=1.55.

Introducing trade‐off to our model allows us to derive the ESS senescence rate. In our model the senescence rate ESS is determined by each species life‐history (G, M, and $x_{s}$) and the trade‐off shape parameters α and β. Higher values of α and β steepen early improvements in each trait but diminish gains near their biological limits ($R_{\min}$ and $\zeta_{\max}$), determining the trade‐off shape. In our example species the ESS is more sensitive to α than β (see Figure S1 in Appendix S1). We set β=1 and fitted α to the empirical species parameterizations assuming the senescence—reproduction trade‐off is at equilibrium. This exercise serves as a sanity check making sure that the parameterization of the trade‐off can fit data from natural populations. We get relatively close values of the fitted α (between 1.02 and 1.55) to the tested mammals, hinting at common biological limitations underlying the trade‐offs shape (Figure 5). Under our assumptions, $\zeta_{\max}$ do not affect the ESS (see Appendix S1). Instead, it is determined by the fitted α given the trade‐off includes the current species reproduction rate. We get reasonable values of species $\zeta_{\max}$ (humans—0.36, whales—0.37, yellow baboons—0.80, and lions—1.15) now hinting at differences in biological limitations on reproduction rate. Even under trade‐off, the ESS senescence rate can be close to zero (e.g., Appendix S1 Figure S1—human and killer whale where β=1, α=2,3). Repeating this analysis with larger dataset of close species at equilibrium can shed light on the shape of the trade‐off between senescence and reproduction rates. This proof of concept should not be used to draw firm conclusions on the exampled species or the shape of the trade‐off in mammals, but rather highlight the need for further research.

## 4 Discussion

In this work we considered senescence evolution by genes that affect mortality from birth, consistent with the current understanding of senescence as a process of damage accumulation. We derived new equations to estimate the selection pressures, considering the biological and environmental parameters. From that, we were able to explain several phenomena that are not predicted by classical theory (Charlesworth 2000). Our main model predictions are the possibility of very low rates of senescence (negligible senescence) evolving through positive feedback, and a common negative association between potential internal risk and senescence rate. We studied the conditions under which these predictions are expected.

Under our model assumptions, slower senescence rate does not necessarily cause weaker selection on further slowing of senescence rate. Rather, a positive feedback loop in senescence evolution can arise, leading to negligible senescence. We demonstrated that selection for negligible senescence increases with late reproduction start‐age, low external mortality (in populations where reproduction rate is density dependent), and slow reproduction rate (in populations where external mortality is density dependent). Potential mortality risk from internal damage increases selection for negligible senescence, except in a range of model parameters where reproduction rate is density dependent (see Table 1, exact solution in Appendix S1). However, when the biological limitations on reducing potential mortality risk from internal damage are weak, senescence would evolve. This explains the puzzle of negligible senescence in organisms with high potential mortality risks from internal damage. Examples include species of bats and birds experiencing extreme oxidative and temperature pressures due to flight (Munshi‐South and Wilkinson 2010; Hickey et al. 2012). Available data on flightless birds, aging faster compared to their flying close relatives supports this hypothesis, considering even exceptions where aerobic capabilities were maintained (Hickey et al. 2012; Pomeroy 1990; Holmes and Ottinger 2003; Ikemoto et al. 2020); Species of moles where low oxygen environment also increase oxidative stress (Andziak et al. 2006); Ant queens exhibiting extreme reproduction rate reducing resource allocation to self‐preservation (von Wyschetzki et al. 2016; Kramer et al. 2021). Our model suggests that in these cases, biological limitations prevent a reduction of the potential mortality risk from internal damage, G, leading to increasingly strong selection (positive feedback) pushing for negligible senescence. This is also an explanation to Peto's paradox, the lack of correlation between body size and cancer rate (Caulin and Maley 2011). Peto's paradox is thoroughly explained by modeling cancer preventing mechanisms and the increased selection on them resulting from large body size and correlated traits (Nunney 1999; Brown et al. 2015; Boddy et al. 2015). Such models give important predictions on the biological and environmental sources of cancer and in what cells we expect cancer to occur (Nunney 2013; Roche et al. 2013). Here instead of modeling mechanisms of cancer prevention specifically, we assume body size is part of the potential risk from internal damage, and cancer preventing mechanisms are part of the factors determining senescence rate (preventing accumulation of mutations).

More generally, our model offers a new explanation for the observed negative correlation between the Gompertz—Makeham parameters, R and G (Strehler and Mildvan 1960), known as Strehler—Mildvan correlation. We find that higher potential mortality risk from internal damage usually leads to increased selection against senescence (Figures 2A,B,D,E and 3C,D). The Strehler—Mildvan correlation has been explained by either an underlying process of damage accumulation with hidden parameters that add interactions between the Gompertz—Makeham model parameters (Strehler and Mildvan 1960; Golubev 2019; Gavrilov and Gavrilova 1991) or by a sensitivity of the model parameters estimation to the stochasticity of mortality data sampling (Burger and Missov 2016; Tarkhov et al. 2017). Here we suggest evolutionary dynamics as another possible cause for the correlation. Our model suggests that such correlation is predicted in the parameter range where negative feedback in the evolution of the parameters exists (Figure 4). A comparative study within a family of species with fitting parameter space can test this model prediction (see (Nunney 2013), for long term study within guppies). Biological limitations and trade‐offs may prevent further senescence rate reduction, even when positive feedback is expected for senescence (Figure 5).

In our model we assumed a Gompertz—Makeham mortality function, and considered the evolution of its parameters. We further considered co‐evolution of the model parameters, focusing, for simplicity, on mutations that can affect parameters independently. We thus derive the observed negative association between G and R (Strehler and Mildvan 1960) from evolutionary dynamics without assuming the correlation in the parameterization of the model. Exploring mutations that change two or more parameters is an interesting topic for future research. To exemplify our results, we used data from natural populations of mammals (Jones et al. 2014) (Figure 1A). However, a naive fitting of data to our model does not allow quantitative predictions regarding specific species. Rather, we use the data to demonstrate qualitative patterns. For example, in the current analysis we assumed that infant mortality is independent from mortality caused by senescence, and that it ends before constant reproduction begins. For species specific conclusions, our model allows relaxing these assumptions.

Our study stands in agreement with previous works examining the connection between external mortality risk, M, and senescence rate, R (i.e., William's hypothesis) (Abrams 1993; Wensink et al. 2017; Williams et al. 2006). In our model, increasing external risk lowers selection for senescence only under density dependence through reproduction. Our model adds that under density dependence through reproduction positive feedback exists, so that low external mortality and low senescence rate increase selection for one another (Figure 4). Our model also offers a new explanation for the widely observed positive correlation between longevity and reproduction start‐age (Harvey and Zammuto 1985; Partridge and Fowler 1992; Møller 2006). Life‐history theory explains that correlation by assuming trade‐offs between senescence rate and investment in growth rate, or parental care (Kirkwood and Rose 1991), but not in all cases can such trade‐offs be identified (Reznick et al. 2005). In our model, higher reproduction start‐age directly increases selection for senescence retardation.

Our work is part of a family of models, starting with Hamilton's, that considered the evolution of genes affecting senescence. In his model, Hamilton described genes that either change mortality instantaneously or permanently. Abrams (1991) argued that since senescence is irreversible, genes that affect mortality through senescence should do so permanently, see also (Promislow and Tatar 1998). Later works examined alternative effects of senescence manipulating genes (Baudisch 2005; Wachter et al. 2013). These works still considered mutations that are expressed after maturity, but their effect can be non‐linear and include epistasis. These works pointed to the importance of investigating the effect of mutations on early life mortality. Our model examined genes that affect the mortality function from conception and the dynamics between such genes. Like those seminal works, we used fitness estimation despite its sensitivity to ecological dynamics (Benton and Grant 2000). The concept of fitness estimation is a useful simplification in our context, allowing us to focus on the selection for senescence without explicitly modeling complicated dynamics that are poorly understood for most species, namely environmental dynamics, biological evolvability limitations, and life‐history trade‐offs. We show that when a trade‐off is known, the calculation of an ESS is possible (Figure 5).

Our results support the view that the theory of senescence evolution should be expanded to include senescence that occurs before reproduction start‐age, not only as part of a pleiotropic effect, but rather as the beginning of a damage accumulation process. Our study complements the classical view that explains senescence evolution by the timing of genes' effects—where selection on genes that affect mortality decreases only after maturity. We expect that many genes affect senescence, some acting before maturity and others after it (López‐Otín et al. 2023; de Magalhães et al. 2009; Brengdahl et al. 2023), and suggest that both types are required to fully understand senescence evolution. Our work points to areas of research that may advance our understanding of senescence evolution. The effect and timing of mutations that change mortality patterns of species is one such area. Another is the study of species with negligible senescence that are adapted to high levels of potential mortality risk from internal damage. Better understanding of senescence evolution could, we hope, contribute to improvements of preservation, health, and well‐being of different species, including humans (Seluanov et al. 2018; Zhao et al. 2021).

## 5 Methods

Gompertz—Makeham mortality curves and reproduction rate were estimated from data in (Jones et al. 2014) using least squares non‐linear regression from reproduction start‐age. Numerical solutions of the equations were calculated using python 3.11, approximating infinite integrals until the age only 0.0001 of the population survives ($l_{x}=0.0001$), and assuming constant reproduction rate, ζ, from reproduction start‐age. Juvenile mortality was accounted for by factoring it into the reproduction rate: $\zeta =\left(1-l_{x_{s}}+l_{x_{s}}^{*}\right)\bar{m_{x}}$. Where $l_{x_{s}}^{*}$ is the ratio of the population that survived until reproduction start‐age including juvenile mortality and $\bar{m_{x}}$ is the average of the observed reproduction rate. Trade‐off's parameters where optimized using a brute‐force algorithm. Approximate conditions of evolutionary feedback were found by analytically exploring the selection gradients of negligible and non‐negligible senescence (see Appendix S1), and numerically solving the exact condition equations.

## Author Contributions

**Lilach Hadany:** conceptualization (equal), methodology (equal), visualization (equal), writing – original draft (supporting), writing – review and editing (lead). **Darar Bega:** conceptualization (equal), methodology (equal), visualization (equal), writing – original draft (lead), writing – review and editing (supporting).

## Funding

This work was supported by Israel Science Foundation, 2064/18 (L.H.).

## Conflicts of Interest

The authors declare no conflicts of interest.

## Supporting information


**Appendix S1:** ece372988‐sup‐0001‐AppendixS1.docx.

## Data Availability

Python scripts to recreate the figures and fit a Gompertz–Makeham curve for the presented species is available at: https://github.com/DararBega/senescence_evolution.
