# Supplementary material for: Evolution of Senescence by Damage Accumulation That Accelerates With Age Throughout an Organism's Lifespan
Source: Ecol Evol. 2026 Feb 25;16(3):e72988. doi: 10.1002/ece3.72988 (PMC12936436; doi:10.1002/ece3.72988)
Supplement: Supplementary file 1 — Appendix S1: ece372988‐sup‐0001‐AppendixS1.docx. [file ECE3-16-e72988-s001.docx]

**Appendix:**

Derivation of selection equations

Notice that the derivative of Gompertz – Makeham survival with respect to R is:

Given that the survival function follows Gompertz – Makeham: $l_{x}=e^{-\frac{G}{R}(e^{Rx}-1)-Mx}$, we use Euler – Lotka fitness: $1=\int_{x_{s}}^{\infty} e^{-rx}l_{x}m_{x}d_{x}$. Assuming $\frac{dR}{dm_{x}}=0$, we differentiate both sides of the equation with respect to $R$: $0=\int_{x_{s}}^{\infty} (-xe^{-rx}\frac{dr}{dR}l_{x}m_{x}+e^{-rx}l_{x}\frac{G}{R^{2}}(e^{Rx}-Rxe^{Rx}-1)m_{x})d_{x}$. separating $\frac{dr}{dR}$ from the equation we get equation 1: $\frac{dr}{dR}=\frac{\frac{G}{R^{2}}\int_{x_{s}}^{\infty} \left( e^{Rx}-Rxe^{Rx}-1 \right)e^{-rx}l_{x}m_{x}dx}{\int_{x_{s}}^{\infty} xe^{-rx}l_{x}m_{x}dx}$. Notice that the derivative of Gompertz – Makeham survival $l_{x}$ with respect to $R$ contains itself: $\frac{dl_{x}}{dR}=(e^{-\frac{G}{R}(e^{Rx}-1)-Mx})\frac{G}{R^{2}}(e^{Rx}-Rxe^{Rx}-1)= l_{x}\frac{G}{R^{2}}(e^{Rx}-Rxe^{Rx}-1)$. Similarly for fitness estimated with $LRS=\int_{x_{s}}^{\infty} l_{x}m_{x}d_{x}$: after differentiating both sides of the equation with respect to $R$ we get equation 2: $\frac{dLRS}{dR}=\frac{G}{R^{2}}\int_{x_{s}}^{\infty} \left( e^{Rx}-Rxe^{Rx}-1 \right)l_{x}m_{x}dx$.

Euler -Lotka selection under negligible senescence

We calculate the survival function when senescence rate approaches 0: $\lim_{R\to0} l_{x}=e^{-\int_{t=0}^{x} \mu_{t}dt}=e^{-\int_{t=0}^{x} (G+M)dt}=e^{-(M+G)x}$. Therefore, when senescence rate approaches 0 and reproduction rate is constant ($m_{x}=\zeta$), Euler – Lotka selection becomes: $\lim_{R\to0} \frac{dr}{dR}=\frac{-\frac{G}{2}\int_{x_{s}}^{\infty} x^{2}e^{-(r+G+M)x}dx}{\int_{x_{s}}^{\infty} xe^{-(r+G+M)x}dx}$ . Solving the integrals gives: $\lim_{R\to0} \frac{dr}{dR}=\frac{\frac{-\frac{G}{2}(e^{-(r+G+M)x_{s}}(2+2(r+G+M)x_{s}+{(r+G+M)}^{2}{x_{s}}^{2}))}{{(r+G+M)}^{3}}}{\frac{e^{-(r+G+M)x_{s}}(1+(r+G+M)x_{s})}{{(r+G+M)}^{2}}}$ $=\frac{-G(2+2(r+G+M)x_{s}+{(r+G+M)}^{2}{x_{s}}^{2})}{(2+2(r+G+M)x_{s})(r+G+M)}$. From the Euler – Lotka equation when senescence is negligible and reproduction rate is constant ($m_{x}=\zeta$) we get: $1=\int_{x_{s}}^{\infty} e^{-(r+G+M)x}\zeta dx\Rightarrow$ $\frac{1}{\zeta}=\frac{1}{(r+G+M)e^{(r+G+M)x_{s}}}\Rightarrow$ $\zeta x_{s}=(r+G+M)x_{s}e^{(r+G+M)x_{s}}\Rightarrow W(\zeta x_{s})= (r+G+M)x_{s}$. Where $W()$ is the product-logarithm i.e., Lambert W function. Under these assumptions, Euler – Lotka selection with negligible senescence simplifies to: $\lim_{R\to0} \frac{dr}{dR}=-\frac{Gx_{s}(2+2W(\zeta x_{s})+{W(\zeta x_{s})}^{2})}{2W(\zeta x_{s})+2{W(\zeta x_{s})}^{2}}$. Notice that density dependence through external mortality would not influence selection for negligible senescence.

*LRS* selection under negligible senescence

When senescence rate approaches 0 and reproduction rate is constant ($m_{x}=\zeta$), *LRS* selection becomes: $\lim_{R\to0} \frac{dLRS}{dR}=\int_{x_{s}}^{\infty} -\frac{G}{2}x^{2}e^{-(M+G)x}\zeta dx\Rightarrow\lim_{R\to0} \frac{dLRS}{dR}=\frac{-\frac{G}{2}e^{-\left( G+M \right)x_{s}}\left( 2+2\left( G+M \right)x_{s}+\left( G+M \right)^{2}{x_{s}}^{2} \right)}{\left( G+M \right)^{3}}\zeta$.

Assuming the population is stable ($LRS=1$), we solve: $\lim_{R\to0} \frac{dLRS}{dR}=\int_{x_{s}}^{\infty} e^{-(M+G)x}\zeta dx=1\Rightarrow\frac{e^{-(M+G)x_{s}}}{(M+G)}\zeta=1$. Thus, when senescence is negligible, $LRS$ selection on senescence rate simplifies to: $\lim_{R\to0} \frac{dLRS}{dR}=-\frac{G(2+2(G+M)x_{s}+{(G+M)}^{2}{x_{s}}^{2})}{2{(G+M)}^{2}}$. Notice that density dependence through reproduction rate would not influence selection for negligible senescence. Reducing $G$ will increase selection for negligible senescence when $0<\lim_{R\to0} \frac{\partial^{2}LRS}{\partial G\partial R}$, this inequality solution is: $\sqrt{2}M<G, x_{s}<\frac{\sqrt{2G^{2}-M^{2}}-M}{\left( G+M \right)^{2}}$.

Selection gradients of $M$, $G$, $x_{s}$, and reproduction rate

Calculating selection gradients for genes that influence, $M$, $G$, $x_{s}$, and reproduction rate assuming it is constant ($m_{x}=\zeta$): A similar analysis to $R$ gives the selection gradients: $\frac{dr}{dG}=\frac{-\frac{1}{R}\int_{x_{s}}^{\infty} \left( e^{Rx}-1 \right)e^{-rx}l_{x}m_{x}dx}{\int_{x_{s}}^{\infty} xe^{-rx}l_{x}m_{x}dx}$, and $\frac{dLRS}{dG}=-\frac{1}{R}\int_{x_{s}}^{\infty} \left( e^{Rx}-1 \right)l_{x}m_{x}dx$ for $G$; $\frac{dr}{dM}=-1$ (this result is Hamilton’s model for $a=0$), and $\frac{dLRS}{dM}=-\int_{x_{s}}^{\infty} xl_{x}m_{x}dx$ (negative of the generation time) for $M$; $\frac{dLRS}{dx_{s}}=-l_{x_{s}}m_{x_{s}}$ and $\frac{dr}{dx_{s}}=\frac{-e^{-rx_{s}}l_{x_{s}}m_{x_{s}}}{\int_{x_{s}}^{\infty} xe^{-rx}l_{x}m_{x}dx}$ for $x_{s}$ (solved using Leibniz integral rule); $\frac{dLRS}{d\zeta}=\int_{x_{s}}^{\infty} l_{x}dx$, and $\frac{dr}{d\zeta}=\frac{\int_{x_{s}}^{\infty} e^{-rx}l_{x}dx}{\int_{x_{s}}^{\infty} xe^{-rx}l_{x}\zeta dx}$ for $\zeta$. Notice that when $R$ approaches 0, selection remains strong for all values of: $G$ ($\lim_{R\to0} \frac{dr}{dG} =-1$, $\lim_{R\to0} \frac{dLRS}{dG}=-\int_{x_{s}}^{\infty} xe^{-rx}l_{x}m_{x}dx$, which is the negative of the generation time, similar to $\frac{dr}{dM}$ and $\frac{dLRS}{dM}$), $M$ , $x_{s}$ ($\lim_{R\to0} \frac{dLRS}{dx_{s}}=-e^{-(G+M)x_{s}}m_{x_{s}}$, $\lim_{R\to0} \frac{dr}{dx_{s}}=\frac{-e^{-(r+M+G)x_{s}}m_{x_{s}}}{\int_{x_{s}}^{x_{d}} xe^{-(r+M+G)x}m_{x}}$), and $\zeta$.

Table S1: Evolutionary dynamics under Euler – Lotka fitness estimation


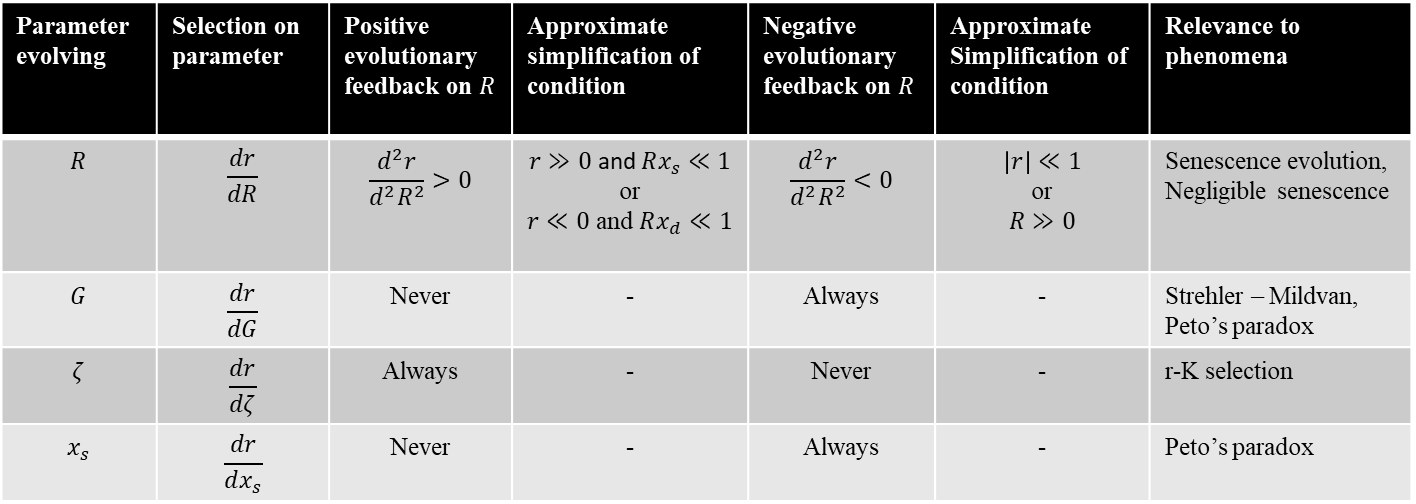


Trade-offs between life-history parameters

In addition to changes that occur in one life-history parameter as a result of another through density dependence, inherent trade-offs between life-history parameter can result from pleiotropy. Classic example is a trade-off between reproduction and senescence rate. Both reducing senescence rate and increasing reproduction rate can have asymptotically increasing costs to one another (but not necessarily). For example, describing this relationship with an equation of a single parameter $z$ that determine where on the trade-off a species is, for $\left( 0\leq z\leq1\leq\alpha,\beta\right)$ and a constant reproduction rate, $\zeta$ , we get:

$$\left\{ \begin{aligned} R={\left( R_{max}-R_{min} \right)z}^{\alpha}+R_{min} \\ \zeta=\left( \zeta_{max}-\zeta_{min} \right)\left( 1-\left( 1-z \right)^{\beta} \right)+\zeta_{min} \end{aligned} \right.$$

Adding such trade-off allows the calculation of the optimal senescence rate: solving $\frac{dr}{dz}=0$, and $\frac{d^{2}r}{d^{2}z^{2}}<0$, if no density dependence is assumed. For density dependent populations there is no optimal senescence rate since, in a stable population $r=LRS-1 =0$. To get an ESS, we need to assume that rare mutations do not change the effect of density dependence (e.g., $\frac{d\zeta^{*}}{dz}=0$, and $\frac{dl_{x}^{*}}{dz}=0$, where $m_{x}^{dd}= \frac{\zeta}{\int_{x_{s}}^{x_{d}} l_{x}^{*}\zeta^{*}dx}$). The invading mutation with reproduction rate $\zeta$ and survival function $l_{x}$ will experience the density dependence effect of the background reproduction, $\zeta^{*}$, and survival, $l_{x}^{*}$. Calculating the ESS for a trade-off between senescence and reproduction rates assuming density dependence through reproduction control $m_{x}^{dd}= \frac{\zeta}{\int_{x_{s}}^{x_{d}} l_{x}^{*}\zeta^{*}dx}$: since $\frac{dR}{dz}={\alpha\left( R_{max}-R_{min} \right)z}^{\alpha-1}$, and $\frac{d\zeta}{dz}=\beta\left( \zeta_{max}-\zeta_{min} \right)\left( 1-z \right)^{\beta-1}$, the selection gradient for $z$ is: $\frac{dLRS}{dz}={\frac{\partial LRS}{\partial R}\frac{dR}{dz}+\frac{\partial LRS}{\partial\zeta}\frac{d\zeta}{dz} =\alpha\left( R_{max}-R_{min} \right)z}^{\alpha-1}\frac{G}{R^{2}}\int_{x_{s}}^{\infty} \left( e^{Rx}-Rxe^{Rx}-1 \right)l_{x}m_{x}^{dd}dx+\frac{\beta\left( \zeta_{max}-\zeta_{min} \right){(1-z)}^{\beta-1}}{\left( \zeta_{max}-\zeta_{min} \right)(1-{(1-z)}^{\beta})+\zeta_{min}}$. An ESS can be found when $\frac{dLRS}{dz}=0$, and $\frac{d^{2}LRS}{d^{2}z^{2}}<0$. Assuming $\zeta_{min},R_{min} =0$, the expression simplifies to: ${\frac{\partial LRS}{\partial z} =\alpha R_{max}z}^{\alpha-1}\frac{G\int_{x_{s}}^{\infty} \left( e^{Rx}-Rxe^{Rx}-1 \right)l_{x}dx}{R^{2}\int_{x_{s}}^{\infty} l_{x}dx}+\frac{\beta{(1-z)}^{\beta-1}}{(1-{(1-z)}^{\beta})}$, which is not dependent on $\zeta_{max}$. We also assume $R_{max}$ is limited by reproduction start-age, $x_{s}$, such that $x_{s}$ cannot be larger than $x_{d}$. The ESS senescence rate becomes dependent only on $G$, $M$, $x_{s}$ and the trade-off parameters $\alpha$ and $\beta$. We explore how $\alpha$ and $\beta$ change the selection gradients for each species estimated parameters (Fig: S1). For our species parameterization the ESS is more sensitive to $\alpha$ than $\beta$. Negligible senescence is possible even under trade-offs when senescence improvement has large reproductive cost (humans where $\alpha=2$ and $\alpha=3$).

Fig S1:


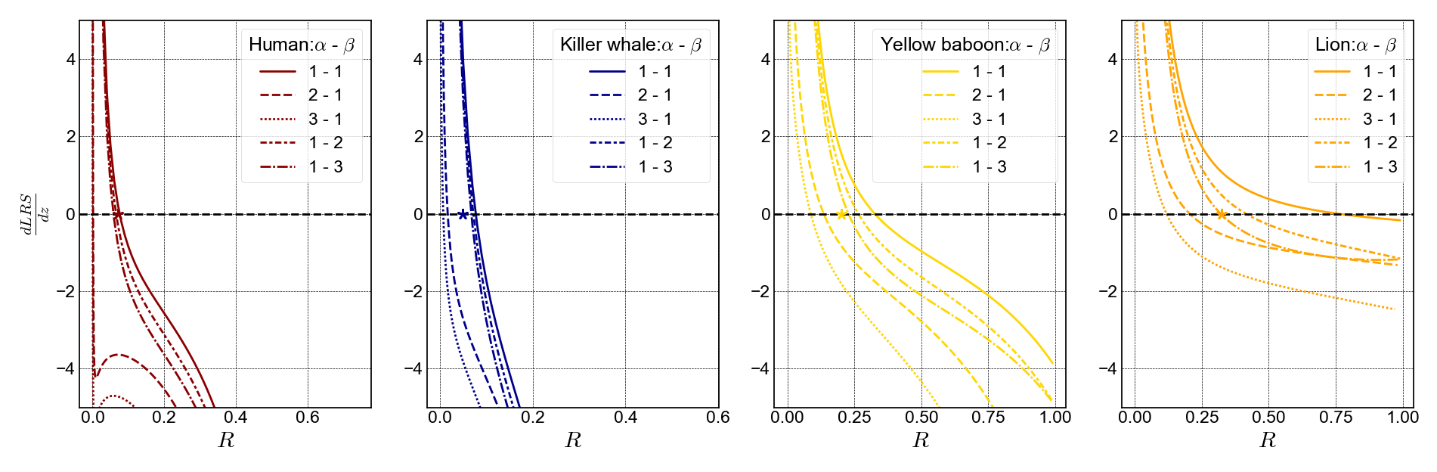


Legend S1:

Selection gradients for senescence rate under density dependence and trade-off with reproduction rate. y-axis is $LRS$ selection. X-axis is senescence rate $R$. Selection gradients of trade-offs with the all permutations of shape parameters $\alpha$ and $\beta$ of values 1, 2, and 3. The Gompertz – Makeham parameters of the different panels fit natural populations of: humans – red color ($G=0.00041$, $M=0.00001$, $x_{s}=13$), whales – blue color ($G=0.00094$, $M=0.00001$, $x_{s}=11$), yellow baboons – yellow color ($G=0.005$, $M=0.03$, $x_{s}=5$), and lion – orange color ($G=0.0025$, $M=0.0522$, $x_{s}=2$). Asterisks represent the species estimated senescence rate $R$.
